# Surgical treatment outcomes of patients with T1-T2 gastric cancer: does the age matter when excellent treatment results are expected?

**DOI:** 10.1186/s12957-018-1388-4

**Published:** 2018-04-16

**Authors:** Rimantas Bausys, Augustinas Bausys, Indre Vysniauskaite, Kazimieras Maneikis, Eugenijus Stratilatovas, Kestutis Strupas

**Affiliations:** 1 Department of Abdominal Surgery and Oncology, National Cancer Institute, Santariskiu str. 1, 08660 Vilnius, Lithuania; 2 Faculty of Medicine, Vilnius University, Ciurlionio str. 21, 03101 Vilnius, Lithuania; 3 School of Medicine, Georgetown University, Washington, D.C., USA; 4 Center of Abdominal Surgery, Vilnius University Hospital Santaros Klinikos, Santariskiu str. 2, 08661 Vilnius, Lithuania

**Keywords:** Gastric cancer, T1, T2, Elderly patients, Early invasion

## Abstract

**Background:**

The proportion of early gastric cancer stages is increasing, as is the incidence of gastric cancer among the elderly population. Therefore, this study was designed to analyze surgical treatment outcomes of T1-T2 gastric cancer in elderly patients.

**Methods:**

A total of 457 patients with T1-T2 gastric cancer who underwent gastrectomy between 2005 and 2015 were enrolled in this retrospective study. Patients were classified into two groups according to age (< 70 years versus ≥ 70 years). Clinicopathological features, surgical treatment results, and clinical outcomes were compared between the groups.

**Results:**

Higher ASA score (ASA 3/4), differentiated cancer, and intestinal-type tumors were more common in elderly patients. Postoperative complication rates were similar between the two groups; however, postoperative mortality rates were significantly higher in the elderly group. Higher ASA score was independently associated with postoperative complications in the elderly group. Furthermore, severe postoperative complications were found as an independent factor associated with higher 90-day mortality rate. Elderly patients had a significantly poorer 5-year overall survival rate. Two surgery-related factors—total gastrectomy and complicated postoperative course—were revealed as independent prognostic factors for poor overall survival in the elderly group.

**Conclusions:**

Despite higher postoperative mortality rate and poorer overall survival results, elderly patients with gastric cancer should be considered for radical surgery. ASA score may be useful for predicting surgical treatment outcomes in elderly patients undergoing surgery for GC and hence assists clinicians in planning treatment strategies for each individual patient.

## Background

Even though the incidence of gastric cancer (GC) has decreased over the past decades, it continues to be a major healthcare problem being the fourth most common malignancy and the second cause of death among all cancers [1]. Treatment strategy of gastric cancer has dramatically changed during the last decades mainly as a result of advances in chemotherapy. However, surgery remains the main and only curative treatment option for GC. Survival rates after curative surgery vary depending on several factors, but the stage of disease and the quality of surgery are the two most important predictors [2]. Best results of surgical GC treatment are achieved when patients undergo surgery while the tumor has only limited invasion. Reports from Asian and Western countries show excellent overall survival (OS) results of patients who underwent surgery for early gastric cancer (pT1) with a 5-year OS rate up to 99% [3–6]. Results for patients with pT2 gastric cancer were also high with a 5-year OS reaching up to 66% [7]. Despite these advancements, such favorable outcomes will be difficult to maintain in the increasing aging population. The increasing lifetime expectancy and improved treatment of age-related chronic diseases have led to a greater number of older patients suffering from GC, who can be potentially cured by surgical resection. While surgery is standard in patients with a stable clinical condition, the indication for operations in elderly patients, especially in those with comorbidities, remains unclear [8–10]. Therefore, identification of factors affecting short-term and long-term surgical treatment outcomes in elderly is essential for treatment personalization and optimization. This is especially significant for a cohort of patients in which excellent outcomes can be expected. Moreover, because early GC stages are relatively rare in the West, only limited data are available to guide treatment decisions for such a population.

The aim of our study was to analyze the differences in surgical treatment outcomes between elderly and non-elderly patients with early invasion (pT1-T2) gastric cancer.

## Methods

### Patients selection

A total of 1654 patients diagnosed with gastric adenocarcinoma underwent surgery at the National Cancer Institute, Vilnius, Lithuania, between 2005 and 2015. One thousand one hundred ninety-seven patients who received gastrectomy for pT3-4 gastric cancer or received neoadjuvant chemotherapy prior to surgery were excluded from the study. The remaining 457 patients who underwent gastrectomy for pT1-2 gastric cancer were included into the final analysis.

### Surgery

Surgical procedures were performed as described in our previous report [3]. Based on tumor location, a total or distal gastrectomy with regional lymph node dissection (D1 or D2) was performed. In all cases, gastrectomy was performed via an open approach. D1 dissection included perigastric lymph nodes as well as the greater and lesser omenta. D2 dissection included the nodes from the D1 dissection plus those along the celiac axis, common hepatic artery, splenic artery and hilum, and the root of the left gastric artery. Resection was considered R0 when microscopically negative resection margin was achieved without macroscopic or microscopic remaining in primary tumor bed. In most cases (119 of 124), reconstruction after a total gastrectomy was performed with esophagojejunostomy using a jejunal loop and side-to-side entero anastomosis (m.Omega). The most common (240 of 320 cases) method of reconstruction after subtotal gastrectomy consisted of an antecolic end-to-side gastrojejunostomy with side-to-side entero anastomosis (m.Balfur) [3]. For the purpose of this study, all patients were divided into elderly (E; ≥ 70 years) and non-elderly (NE; < 70 years) groups according to the age at the time of surgery. Postoperative chemotherapy was recommended to all patients with pT1N+ or pT2N0/N+ gastric cancer.

### Personal characteristics and clinical data

All patient characteristics were obtained from their medical records and prospectively collected database. Tumor stage was coded according to the TNM system as described in the Union Internationale Contre le Cancer/American Joint Committee on Cancer 7th edition. Demographic characteristics included age and sex. Clinicopathological characteristics included smoking status, body mass index (BMI), comorbidities, American Society of Anesthesiology (ASA) score, hospitalization and intensive care unit (ICU) time, type of surgery and lymphanodectomy, length of surgery, blood loss, tumor location, tumor size, differentiation, depth of invasion, lymphovascular invasion, and retrieved and metastatic lymph node count. Postoperative morbidity and mortality were evaluated. All postoperative complications were graded according to Clavien-Dindo classification.

OS analysis was performed. OS was defined as time from surgery to death. Data of survival and date of death were collected from Lithuania’s Cancer register and Lithuania’s death register [3]. The last follow-up was performed on the 31st of December 2016. Two (0.44%) patients were lost during the follow-up period. Mean and median follow-up periods were 52 and 45 months (range from 0 to 142) respectively.

### Statistical analysis

Statistical package SPSS 16.0 (SPSS, Chicago, IL, USA) was used for statistical analysis. Groups were compared by a two-tailed *t* test, one-way ANOVA test, chi-square test, Fisher exact test, or non-parametric tests. All potential risk factors for postoperative mortality and morbidity were included in subsequent multivariate logistic regression analyses. Independent variables associated with postoperative morbidity and mortality were identified. OS analysis was performed by the Kaplan-Meier method. Survival curves were compared by the log-rank test. Multivariate survival analysis was performed using the Cox proportional hazards model (hazard ratio and 95% confidence intervals). In all statistical analyses, a *p* value of < 0.05 was considered to be significant.

## Results

### Clinicopathological characteristic of gastric cancer in NE and E groups

At the time of surgery, 267/457 (58.4%) patients were younger than 70 years. One hundred ninety (41.6%) patients were 70 or older. Table 1 summarizes the clinicopathological findings of the two study groups.

**Table 1:** Comparison of patient characteristics

		Group NE (< 70 years)		Group E (≥ 70 years)		*p* value
		*n*	%	*n*	%	
Sex	Male	150	56.2	111	58.4	0.701
	Female	117	43.8	79	41.6	
Age		58.18 ± 8.86		76.43 ± 4.35		0.001
BMI		26.2 ± 5.72		25.9 ± 4.90		0.700
ASA score	1–2	177	68.3	71	38.6	0.001
	3–4	82	31.7	113	61.4	
Tumor localization (third)	Upper 1/3	51	19.1	26	16.8	0.147
	Middle 1/3	119	44.6	80	42.1	
	Lower 1/3	97	36.3	84	44.2	
Gastrectomy	Total	83	31.1	47	24.7	0.143
	Subtotal	184	68.9	143	75.3	
Lymphanodectomy	D1	13	5.0	18	9.8	0.059
	D2	246	95.0	165	90.2	
Multivisceral surgery	Yes	19	7.3	15	8.2	0.721
	No	241	92.7	168	91.8	
Tumor invasion	T1a	64	24.0	35	18.4	0.217
	T1b	63	23.6	56	29.5	
	T2	140	52.4	99	52.1	
Lymph node metastasis	Yes	101	37.8	74	38,9	0.845
	No	166	62.2	116	61,1	
N categories	N0	166	62.2	116	61.1	0.778
	N1	65	24.3	49	25.8	
	N2	19	7.1	17	8.9	
	N3	17	6.3	8	4.3	
Distant metastasis	Yes	5	1.9	3	1.6	0.999
	No	262	98.1	187	98.4	
Lauren classification	Diffuse	112	42.3	17	21.8	0.001
	Mix	31	11.7	6	8.9	
	Intestinal	121	45.8	63	69.3	
Tumor differentiation grade	G1	27	10.1	25	13.2	0.001
	G2	69	25.8	84	44.2	
	G3	171	64.0	81	42.6	
Tumor size	< 2 cm	88	34.2	56	30.6	0.471
	≥ 2 cm	169	65.8	127	69.4	

Significant differences were found between the groups in terms of physical status (ASA score), histological type of tumor, and tumor differentiation grade. Significantly larger proportion of elderly patients had severe systemic diseases (ASA 3–4) (61.4 vs. 31.7%, *p* = 0.001) and intestinal type tumors according to Lauren classification (69.3 vs. 45.8%, *p* = 0.001). Poorly differentiated tumors were more common in the NE group (G3: 64.0 vs. 42.6%, *p* = 0.001).

### Postoperative morbidity and mortality

The short-term surgical outcomes are shown in Table 2. There was no significant difference in the rate of total, severe (3rd or 4th grade according to Clavien-Dindo classification), or surgical postoperative complications between the two groups, but the rate of medical complications was significantly higher in the E group (9.7 vs. 16.3%, *p* = 0.035). Furthermore, fatal complications which led to postoperative deaths were observed only in elderly patients, and the mortality rate was significantly higher in this group (0 vs. 5.7%, *p* = 0.001). Even higher differences were observed when 30- and 90-day mortality rates were compared. First deaths in NE group were observed between 30th and 90th postoperative days with the 90-day mortality rate reaching 2.6%. During the same time period, mortality rate in the E group increased from 7.4 to 12.6% and remained significantly higher when compared to the NE group.

**Table 2:** Short-term surgical outcomes of NE and E patients who underwent gastrectomy for pT1-T2 gastric cancer

Factors		Group NE (< 70 years)	Group E (≥ 70 years)	*p* value
Operation time: min (mean ± SD; min-max)		146.9 ± 41.58	143.72 ± 44.53	0.432
Blood loss: ml (mean ± SD; min-max)		171.82 ± 137.11	169.50 ± 136.50	0.879
ICU stay: days (mean ± SD; min-max)		1.24 ± 1.24	2.23 ± 6.30	0.014
Postoperative hospital stay: days (mean ± SD; min-max)		13.07 ± 5.86	14.05 ± 8.24	0.145
Dissected lymph nodes (mean ± SD)		22.18 ± 10.28	19.50 ± 9.43	0.005
Curability	R0	259 (97.0%)	182 (95.8%)	0.484
	R1,2	8 (3.0%)	8 (4.2%)	
Postoperative complications: *n* (%)		55/267 (20.5%)	51/190 (26.8%)	0.144
Surgical complications		29/267 (10.8%)	20/190 (10.5%)	0.909
Anastomotic leakage		2/267 (0.7%)	5/190 (2.6%)	0.133
Postoperative bleeding		6/267 (2.2%)	4/190 (2.1%)	0.999
Peritonitis/ intraabdominal abscess		5/267 (1.8%)	3/190 (1.5%)	0.999
Ileus		3/267 (1.1%)	0/190 (0.0%)	0.269
Incisional surgical site infection and (or) eventration		5/267 (1.8%)	4/190 (2.1%)	0.999
Postoperative pancreatitis		5/267 (1.8%)	2/190 (1.0%)	0.704
Pancreatic/biliary/enterocutaneuos fistula		3/267 (1.1%)	2/190 (1.0%)	0.999
Medical complications		26/267 (9.7%)	31/190 (16.3%)	0.043
Cardiac insufficiency		0/267 (0.0%)	3/190 (1.5%)	0.071
Pneumonia		11/267 (4.1%)	4/190 (2.1%)	0.292
Sepsis		0/267 (0.0%)	3/190 (1.5%)	0.071
PATE		0/267 (0.0%)	2/190 (1.0%)	0.172
Other		15/267 (5.6%)	19/190 (10.0%)	0.102
Clavien-Dindo	1–2	39 (14.6%)	30 (15.8%)	0.195
	3–4	16 (6.0%)	10 (5.3%)	
Postoperative mortality: *n* (%)		0 (0.0%)	11 (5.7%)	0.001

### Risk factors for postoperative complications in NE and E groups

At univariate analysis, ASA III/IV (*p* = 0.006), total gastrectomy (*p* = 0.022), and multivisceral surgery (*p* = 0.031) were identified as factors that were associated with postoperative complications in the E group (Table 3). At multivariate analysis, only ASA III/IV was independently associated with postoperative complications (OR = 6.47; 95% CI 2.09–20.06, *p* = 0.021).

**Table 3:** Univariate analysis of risk factors for postoperative complications in NE and E groups

		Group NE (< 70 years)	%	*p* value	Group E (≥ 70 years)	%	*p* value
Sex	Male	30/150	20.8	0.879	30/111	27.0	0.999
	Female	25/117	21.4		21/79	26.6	
BMI	< 30	30/130	23.1	0.999	9/30	30.0	0.814
	≥ 30	11/47	23.4		24/91	26.4	
ASA score	1–2	33/177	18.6	0.144	11/71	15.5	0.006
	3–4	22/82	26.8		39/113	34.5	
Gastrectomy	Total	22/83	26.5	0.141	19/47	40.4	0.022
	Subtotal	33/184	17.9		32/143	22.4	
Tumor localization (third)	Upper 1/3	15/51	29.4	0.077	11/26	42.3	0.157
	Middle 1/3	24/119	20.2		19/80	23.8	
	Lower 1/3	16/97	16.5		21/84	25.0	
Multivisceral surgery	Yes	2/19	10.5	0.381	8/15	53.3	0.031
	No	53/241	22.0		42/168	25.0	
Lympha-nodectomy	D1	5/13	38.5	0.157	5/18	27.8	0.999
	D2	50/246	20.3		46/165	27.9	
Retrieved lymph nodes (LN)	≤ 15 LN	16/52	30.8	0.058	17/61	27.9	0.860
	> 15 LN	39/211	18.5		33/127	26.0	

In the NE group, none of the analyzed factors were significantly associated with total number of postoperative complications, but tumor localization in the upper third was associated with severe postoperative complications (≥ 3 grade according to Clavien-Dindo), 14.9 vs. 4.3%, *p* = 0.014.

### Risk factors for postoperative mortality in NE and E groups

While there were no deaths in the NE group during first 30 postoperative days, factors associated with 90-day mortality rate were analyzed. At univariate analysis, ASA 3/4 (*p* = 0.034) and tumor localization in the upper third (*p* = 0.013) were associated with 90-day mortality in the NE group.

In the E group, univariate analysis revealed ASA 3/4 (*p* = 0.038) and multivisceral surgery (*p* = 0.017) as factors associated with death during the first 90 postoperative days. With multivariate analysis, only severe complications during hospitalization were found as independent factors associated with higher 90-day mortality rate (OR = 12.82; 95% CI 1.01–169.21, *p* = 0.049).

**Fig. 1:**
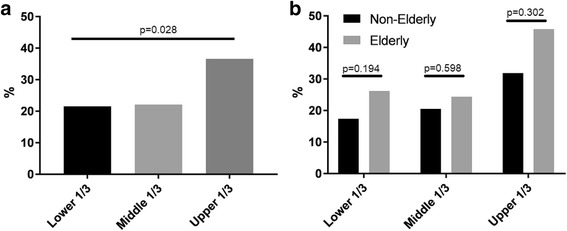
Postoperative complications after surgery for different localizations gastric cancer. **a** Postoperative complications after surgery for different localization cancer in the entire study cohort. **b** Comparison of postoperative complications between non-elderly and elderly groups according to tumor localization

Furthermore, we found the higher rate of postoperative complications after surgery for upper third tumors in the entire study cohort (Fig. 1a). Analysis of specific complications showed that surgery for upper third cancer leads to higher rate of anastomotic insufficiency (upper third 5/77 (6.4%) vs. middle or lower third 2/380 (0.5%), *p* = 0.001). However, we did not found a significant difference in postoperative complications rate after surgery for upper third tumors between the NE and E groups (Fig. 1b).

### Survival analysis

**Fig. 2:**
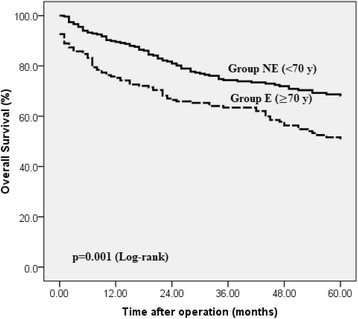
Overall survival after surgery for T1-T2 gastric cancer in elderly and non-elderly groups. Non-elderly (NE) patients have significantly higher 5-year overall survival rate after surgery for T1/T2 gastric cancer

Five-year OS rate after surgical treatment of pT1/2 gastric cancer reached 60.8% in our study cohort. Non-elderly patients had significantly higher OS rate, 67.8 vs. 51.1%, *p* = 0.001 (Fig. 2).

Kaplan-Meier analysis revealed total gastrectomy, multivisceral surgery, T2 tumor invasion, lymph node and distant metastasis, lymphovascular invasion, and tumor size ≥ 2 cm to have a negative effect upon OS in both study groups (Table 4). In the multivariate analysis, male gender, lower BMI, deeper tumor invasion, and diffuse tumor type were found to be independent prognostic factors of OS results in the NE group. However, none of these factors showed a significant impact in the E group. Only total gastrectomy and postoperative complications were independent prognostic factors in this subgroup of patients.

**Table 4:** Factors affecting overall survival in NE and E patient groups. Kaplan-Meier (univariate) and Cox regression (multivariate) analysis

		Group NE (< 70 years)			Group E (≥ 70 years)		
		Univariate analysis		Multivariate analysis	Univariate analysis		Multivariate analysis
		5-year OS (%)	*p* value	HR (95% CI); *p* value	5-year OS (%)	*p* value	HR (95% CI); *p* value
Sex	Male	66.0	0.263	5.24 (1.26–21.79); 0.023	46.8	0.094	0.64 (0.16–2.50); 0.528
	Female	70.1			57.0		
BMI	< 30	65.5	0.202	7.75 (1.37–43.72); 0.020	61.5	0.678	3.71 (0.69–19.69); 0.124
	≥ 30	73.9			56.7		
ASA score	1–2	70.6	0.190	2.01 (0.59–6.80); 0.261	59.2	0.052	1.88 (0.53–6.70); 0.326
	3–4	64.6			46.0		
Lymphano-dectomy	D1	76.9	0.745	1.21 (0.74–1.86); 0.981	38.9	0.172	3.93 (0.37–40.80); 0.251
	D2	68.7			53.3		
Retrieved lymph nodes	≤ 15	73.1	0.310	1.15 (0.24–5.44); 0.854	45.9	0.443	2.89 (0.60–13.93); 0.186
	> 15	67.3			53.5		
Gastrectomy	Total	49.4	0.001	2.32 (0.59–9.14); 0.227	34.0	0.012	10.14 (2.15–47.77); 0.003
	Subtotal	76.1			56.6		
Multivisceral surgery	Yes	47.4	0.006	1.93 (0.38–9.78); 0.425	53.6	0.019	22.83 (0.39–132.69); 0.131
	No	70.5			33.3		
Postoperative complications	Yes	72.7	0.425	1.57 (0.38–6.43); 0.525	21.6	0.001	3.94 (1.05–14.82); 0.042
	No	66.5			61.9		
Tumor invasion	T1a	89.1	0.001	5.81 (1.14–29.69); 0.034	65.7	0.010	4.29 (0.39–46.19); 0.229
	T1b	85.7			62.5		
	T2	50.0			39.4		
Lymph node metastasis	LNM+	80.7	0.001	2.02 (0.66–6.18); 0.215	36.5	0.001	0.54 (0.09–3.32); 0.512
	LNM-	46.5			60.3		
Distant metastasis	M1	0.0	0.001	4.30 (0.31–58.14); 0.271	51.9	0.001	2.20 (0.09–53.74); 0.628
	M0	69.1			0.0		
Tumor localization (third)	Upper	49.0	0.001	2.02 (0.36–11.21); 0.419	34.6	0.298	1.04 (0.29–3.74); 0.947
	Middle	71.4			55.0		
	Lower	73.2			52.4		
Lauren classification (type)	Diffuse	60.7	0.086	9.72 (1.73–54.53); 0.010	59.0	0.147	4.82 (0.99–17.41); 0.929
	Mix	64.5			68.8		
	Intestinal	74.4			44.4		
Tumor differentiation grade	G1	81.5	0.037	2.21 (0.29–16.93); 0.443	68.0	0.074	3.78 (1.03–24.39); 0.910
	G2	76.8			42.9		
	G3	62.0			54.3		
Ulceration	UL+	67.1	0.901	1.51 (0.55–4.15); 0.415	56.5	0.583	2.37 (0.66–8.51); 0.184
	UL-	69.6			50.7		
Lymphovascular invasion	LV+	45.8	0.001	2.00 (0.61–6.51); 0.250	42.9	0.005	2.50 (0.53–11.72); 0.245
	LV-	79.1			60.9		
Signet ring cell carcinoma	SRC+	67.7	0.601	2.49 (0.64–9.65); 0.186	71.4	0.354	3.92 (0.12–11.99); 0.433
	SRC-	60.6			50.9		
Tumor size	< 2 cm	84.1	0.001	1.27 (0.37–4.31); 0.693	66.1	0.010	4.09 (0.80–20.95); 0.091
	≥ 2 cm	61.5			45.7		

## Discussion

With the population in Europe and Lithuania aging, the proportion of patients with gastric cancer who are elderly at the time of diagnosis is increasing [11]. Naturally, the definition of elderly patients varies in different studies with cutoff values ranging from 65 to 70 years to 75 or even 80 years [11–13]. However, average lifetime expectancy in Lithuania is lower compared to most developed Western or Asian countries. According to WHO report, the average lifetime expectancy in Lithuania in 2015 was 73.6 years. Therefore, we used 70 years as the threshold. With this selection, the elderly group amounted to 41.6% of the total cohort in this study.

Several aspects should be discussed with respect to the clinicopathological differences between the groups of our study. We found two distinct histological features. First, the intestinal type of tumor was more often found in the E group. These results support previously published data [14–17]. Higher frequency of intestinal gastric cancer with increasing age has been explained by studies, which showed intestinal GC development in areas of the stomach where intestinal metaplasia occurs. With chronic atrophic gastritis, a gradual change from normal mucosa to intestinal-type mucosa takes many years to develop. The process peaks in elderly age contributing to an increased risk of intestinal gastric cancer [18, 19]. The second difference that we noted was that the incidence of differentiated cancer (well or moderately differentiated adenocarcinoma) was significantly higher in the E group. Higher incidence of differentiated tumors supports the hypothesis that gastric cancer in an elderly age develops first as a differentiated type of tumor and only later, probably due to long-lasting chronic gastritis, these tumors progress to an undifferentiated one. Both of these unique findings emphasize the major role of chronic gastritis in the GC development and progression in elderly individuals.

There was no significant difference in the overall and surgical postoperative complication rate between the NE and E groups. These results oppose studies published in the early 2000s, where age was shown to be a major predictor of postoperative complications or mortality [20, 21]. Instead, it supports results of more recent studies, demonstrating that advances in surgical and anesthesiological techniques have reduced surgical complications and consequently improved short-term surgical outcomes in elderly patients [22, 23]. One of the differences between gastric cancer surgeries between the different age groups is lymph node dissection, which is usually limited in the elderly. Although the rate of D1 or insufficient (≤ 15 lymph nodes) lymphanodectomy was not significantly higher in the E group, we did find a significant difference when the average number of retrieved lymph nodes was compared between the groups (22.18 vs. 19.50, *p* = 0.005). However, in contrast to most Western and Japanese reports [24–26], we failed to show the association between limited lymphanodectomy and lower postoperative morbidity or mortality. Perhaps, this discrepancy can be explained by the retrospective design of our study and the fact that limited lymphanodectomies were performed for patients with a high morbidity risk.

Other well-known surgical risk factors for postoperative morbidity are the extent of gastric resection (total or subtotal) and multivisceral resection [20, 25, 26]. Our results confirmed the association between these two factors and higher postoperative complication rates in the E group. Generally, according to the principles of surgical oncology, subtotal gastrectomy may be carried out if an adequate proximal margin can be achieved. However, the requirements for proximal margin vary between different guidelines. Our study cohort included only patients with early invasion (pT1-T2) GC in which the recommended proximal margin is 5–8 cm according to the European Society for Medical Oncology guidelines. On the other hand, the Japanese gastric cancer treatment guidelines indicate only 3–5 cm in cases of T2 tumors and 2 cm in cases of T1 tumors. In our opinion, less radical resection margins are preferred to avoid a total gastrectomy, especially for patients with high risk of postoperative complications.

Even though the rate of total complications was comparable between the study groups, we found significantly higher number of medical complications in the E group. Furthermore, 5.7% of elderly patients died after postoperative complications occurred, while there were no deaths in the NE group. Hayashi et al. reported similar results [27], in which elderly patients with complicated postoperative courses after the gastrectomy had higher mortality rates. Consequentially, elderly patients should receive special attention if postoperative complications occur. Precise surgical risk assessment prior to surgery is crucial when an optimal treatment strategy is being determined for individual patients. As expected, the number of patients with an ASA score of 3 or 4 was significantly higher in the E group (31.7 vs. 61.4%). In contrast to other reports [24, 28–30], we found that higher ASA score is an independent risk factor for postoperative complications in the E group. Moreover, we correlated a higher ASA score to an increase in 90-day mortality rates in the NE group. Therefore, evaluating the physical status of patients using the ASA classification is a reliable tool in predicting the short-term outcomes in both the E and NE groups.

In determining the appropriate treatment for each individual, patient prognosis should also be considered before turning to surgery. Many studies have specifically compared the long term-outcome of GC in elderly patients with non-elderly. Most of these have confirmed that the prognosis for elderly patients was poorer [19, 31, 32]. Our results are consistent with such reports as we found a significantly higher 5-year overall survival rate in the NE group. Additionally, after performing a multivariate Cox regression analysis, we determined different prognostic factors for poor OS results in the two groups. These results indicate different pathways of poor long-term outcomes in non-elderly and elderly patients. In the NE group, two subgroups of determinants were significant. First, patient characteristics—male gender and lower BMI—were correlated to a poorer prognosis. Male gender and worse prognosis were previously shown by Sato et al. [33] and were most likely linked to a shorter lifetime expectancy in the male population. Lower BMI has been known to be associated with specific respiratory postoperative complications and respiratory causes of death, complicating survival rates [11]. The second subgroup corresponds to tumor-related factors, specifically deeper tumor invasion and diffuse tumor type. Similarly, Ikoma and colleagues [7] have published data in which T2 invasion was found to be an independent risk factor for shorter survival. Deeper invasion of the tumor was associated with more advanced disease and to its link of significantly higher rates of lymph node metastasis. Histological tumor type according to Lauren classification also influences OS results. Series of reports have shown that diffuse-type cancer has a worse prognosis [34, 35] as was confirmed by our results.

Contrary to the predictive factors determined in the NE group, we failed to find any patient or tumor-related links to poor OS results in the E group. Although total gastrectomy and postoperative complications were two independent risk factors of decreased OS in the E group, both of them were related to the surgery itself. We hypothesize that with a total gastrectomy, poor OS results are due to the prolonged duration of surgery and more extensive intraabdominal manipulations, which can result in higher risks of postoperative complications and deaths in early postoperative period. Furthermore, Mantovani et al. [36] suggested that prolonged inflammatory responses could promote the proliferation and survival of cancer cells. Based on this report, the association of poor long-term outcomes and postoperative complications can be explained by the idea that residual cancer cells stimulated by inflammatory responses, caused by postoperative complications, result in proliferation and metastasis of cancer cells. Additionally, postoperative complications are associated with adjuvant chemotherapy omission and treatment delays resulting in postpone of residual cancer treatment. Therefore, postoperative complication prevention may play a major role in improving not only short-term but also long-term results in elderly patients with GC.

## Conclusion

Elderly patients with early invasion of GC have a similar risk of postoperative complications as the non-elderly population. However, they should receive special attention in cases of complicated postoperative courses because mortality rates in the elderly are significantly higher.

Our study suggests that the ASA score may be useful in predicting postoperative complications in elderly patients undergoing surgery for GC. It offers clinicians another tool in optimizing treatment strategies. Finally, our results suggest that radical surgery with at least limited lymph node dissection should be considered, even for elderly patients.
